# Efficacy and safety of moxibustion in patients with chronic prostatitis/chronic pelvic pain syndrome: A systematic review protocol

**DOI:** 10.1097/MD.0000000000015678

**Published:** 2019-05-17

**Authors:** Qianan Cao, Xu Zhou, Jianrong Chen, Yuting Zhong, Haifeng Zhang, Qi Ao, Meilu Liu, Heyun Nie, Weifeng Zhu, Yong Fu

**Affiliations:** a Jiangxi University of Traditional Chinese Medicine, Nanchang, Jiangxi; b Department of Endocrinology, Second Affiliated Hospital, Chongqing Medical University, Chongqing; c Department of internal medicine, Jiangxi Cancer Hospital, Nanchang, Jiangxi, China.

**Keywords:** chronic prostatitis/chronic pelvic pain syndrome, moxibustion, protocol, systematic review

## Abstract

**Background:**

Chronic prostatitis/chronic pelvic pain syndrome (CP/CPPS) is a common urogenital disease. Moxibustion is a complementary treatment option for CP/CPPS. This systematic review will assess the efficacy and safety of moxibustion as a sole or add-on therapy for CP/CPPS.

**Methods:**

We will retrieve randomized controlled trials (RCTs) of moxibustion for CP/CPPS from the following databases: PubMed, EMBASE, Cochrane Central Register of Controlled Trials, VIP, Chinese Biomedical Database, China National Knowledge Infrastructure Database, Wanfang Data, Chinese Medicine Database System, Google Scholar, Clinicaltrials.gov, and China Clinical Trial Registry from their inception to March 9, 2019, without language restrictions. RCTs comparing moxibustion with active drugs or moxibustion + drugs with these same drugs alone will be included. Primary outcomes will be the change in the total score of the National Institutes of Health's Chronic Prostatic Inflammatory States Index (NIH-CPSI) after moxibustion treatment. Secondary outcomes will include the scores of the individual NIH-CPSI domains, response to treatment of CP/CPPS, leucocyte and phosphatidylcholine corpuscle count in prostatic fluid, incidence of adverse events (AEs), and incidence of moxibustion-related AEs. The Cochrane risk of bias tool will be used for evaluating the risk of bias of individual trials. Heterogeneity will be detected by the Cochran Q test and I-square test. A random-effects model will be used to pool data in the meta-analysis. Risk ratio and weighted or standardized mean difference will be used as the effect measures. Three sets of subgroup analyses will be performed to explore the sources of heterogeneity. Where appropriate, we will assess the likelihood of publication bias based on funnel plots and quantitative tests.

**Results:**

This study will produce the systematic review evidence regarding moxibustion for treating CP/CPPS based on current RCTs.

**Conclusion:**

This study will provide a clear basis for understanding the efficacy and adverse reactions of moxibustion treatment for CP/CPPS.

**PROSPERO registration number:**

CRD42019121338.

## 1 Introduction

### 1.1 Description of the condition

Chronic prostatitis/chronic pelvic pain syndrome (CP/CPPS) is a pelvic floor dysfunction in men, which commonly manifests as urogenital pain, lower urinary tract symptoms, sexual dysfunction, and psychological issues.^[1]^ In America, CP/CPPS affects over 2 million men, with a prevalence estimated at 1.8% in 2013.^[2]^ In China, a national survey showed that the prevalence of CP/CPPS was 4.5% in 2009.^[3]^ The routine pharmacological interventions for CP/CPPS include antibiotics, α-adrenergic antagonists, and analgesics.^[4–6]^ However, these drugs have use limitations, due to their poor efficacy, antimicrobial resistance, frequent side effects (e.g., sleeplessness, daytime fatigue, vomiting, nausea, and skin itching), and financial burden.^[7]^ The medical expense of CP/CPPS was $1397 per patient in America and $1151 per patient in China.^[4]^

### 1.2 Description of the intervention

Moxibustion is a critical intervention in traditional Chinese medicine (TCM). The ancient classics that first record moxibustion are “Moxibustion Therapy on the Eleven Meridians of Yin and Yang” and “Moxibustion Therapy on the Eleven Meridians of Legs and Arms” which were written 2000 years ago.^[8]^ Moxibustion delivers heat stimulation at acupoints on the body surface by burning moxa leaves or moxa floss, with treatment effects in various diseases.^[9,10]^

There are 2 types of moxibustion in clinical practice: direct moxibustion, which directly applies heat simulation to the skin, and indirect moxibustion, which insulates heat simulation by various materials (e.g., ginger, garlic, or salt).^[11]^ Moxibustion is widely used in East Asia and is recommended for treating chronic inflammation and pain diseases, including osteoarthritis, lumbago, allergic asthma, dysmenorrhea, and CP/CPPS, by the World Health Organization Advisory Committee on Acupuncture and Moxibustion.^[12]^

### 1.3 How the intervention might work

The analgesic and anti-inflammatory effects of moxibustion for CP/CPPS have been proved by several animal and human studies. CP/CPPS is mainly caused by a sedentary lifestyle and irregular diet, which cause chronic congestion and swelling in the prostate.^[13]^ The heat stimulation of moxibustion could improve the microcirculation and metabolism of the prostate, thereby eliminating chronic congestion and swelling by dilating the blood vessels and improving blood flow.^[14]^ Another important cause of CP/CPPS is pathogens and necrotic tissue, which results in the increased expression of pro-inflammatory cytokines, such as interleukin-1β and tumor necrosis factor-α, which stimulates expression of other chemokines (e.g., macrophage inflammatory protein-1α and monocyte chemoattractant protein-1), resulting in damage to the prostate tissue.^[15,16]^ The mechanism underlying the anti-inflammatory effects of moxibustion may involve reduced expression of pro-inflammatory cytokines and chemokines.^[17]^

### 1.4 Why it is important to perform this review

Because of the characteristics of potential efficacy, convenience, inexpensive cost, and safety, moxibustion has potential as a complementary intervention for long-term healthcare of patients with CP/CPPS. Some randomized controlled trials (RCTs) have been performed to investigate the effects of moxibustion on CP/CPPS, but their results have been inconsistent. To date, there is no systematic review-based evidence regarding moxibustion for CP/CPPS. Thus, to obtain convincing evidence for clinical practice, we will systematically review the published RCTs and compare the efficacy and safety of moxibustion with drug/non-drug interventions on CP/CPPS.

### 1.5 Objective

The aim of this systematic review is to retrieve and include published RCTs, systematically, to evaluate the efficacy and safety of moxibustion in CP/CPPS patients.

## 2 Methods

### 2.1 Study registration

This systematic review was registered in PROSPERO (No: CRD42019121338). The reporting of this protocol is structured according to the Preferred Reporting Items for Systematic Reviews and Meta-Analyses Protocols (PRISMA-P) guidelines.^[18]^

### 2.2 Criteria for including studies in this review

#### 2.2.1 Types of studies

Parallel and crossover RCTs will be eligible for inclusion. Semi-randomized controlled trials and patient/clinician preference-based trials will be excluded.

#### 2.2.2 Types of participants

Eligible patients will be adult patients who meet the diagnostic criteria for category III chronic prostatitis of National Institute of Health (NIH).^[19]^ Generally, patients who suffer pain symptoms in the prostate region or urinary tract symptoms lasting ≥3 months within a 6-month period, without urinary tract infection, can be diagnosed with CP/CPPS. Studies that enrolled patients with acute bacterial prostatitis, benign prostatic hyperplasia or other prostate diseases, or patients with severe heart disease, hepatic and kidney dysfunction, severe mental disease, cancer, or other serious diseases will be excluded.

### 2.3 Types of interventions

#### 2.3.1 Experimental interventions

Eligible interventions in the experimental group include direct or indirect moxibustion. Moxibustion is defined as a treatment that uses heat generated by ignited moxa to stimulate acupoints to achieve treatment effects. Direct moxibustion includes gentle moxibustion, circling moxibustion, pecking sparrow moxibustion, and heat-sensitive moxibustion. Indirect moxibustion includes sandwiched moxibustion (e.g., ginger, garlic, salt, aconite, etc), thunder-fire moxibustion, Taiyi miraculous moxa roll, and navel moxibustion. The combination of medical therapies, such as antibiotics, plant preparations, alpha-receptor blockers, m-receptor blockers, non-steroidal drugs, or Chinese herbal medicine, or lifestyle interventions, will be allowed. Moxibustion that is not based on moxa (e.g., burning rush moxibustion and crude herb moxibustion) and moxibustion that causes trauma (e.g., scar moxibustion) will be excluded. Other acupoint stimulation methods, such as acupuncture, acupressure, massage, cupping, scrapping therapy, bloodletting therapy, acupoint injection, acupoint catgut embedding therapy, and so on, will also be excluded.

#### 2.3.2 Comparator interventions

Eligible comparisons will include moxibustion + drugs versus the same drugs alone, and moxibustion versus drugs. Placebo (sham moxibustion) and lifestyle interventions will be allowed in either group. The following treatment will not be allowed as the control: prostate massage, biofeedback therapy, surgical treatment, and acupoint simulation therapies (e.g., moxibustion, acupuncture, acupressure, cupping, scrapping, bloodletting, acupoint injection, and acupoint catgut embedding therapy).

### 2.4 Types of outcome measures

#### 2.4.1 Primary outcomes

Changes in the total score of the NIH Chronic Prostatitis Symptom Index (NIH-CPSI) scale. The NIH-CPSI includes 9 items, with a total score range from 0 to 43 points. A higher score indicates more severe clinical symptoms.

#### 2.4.2 Secondary outcomes

(1) Each individual domain score of the NIH-CPSI, including pain and discomfort (0–21 points), urination symptoms (0–10 points), and quality of life (0–12 points).^[20]^
(2) Response to treatment of CP/CPPS, assessed by the criteria in the *Guiding Principles of Clinical Research on New Drugs of Traditional Chinese Medicine* (2002 edition).^[21]^ It is divided into 4 classifications, that is, complete response, significant response, partial response, and ineffective response, based on the proportion of reduction in the score of a symptom scale specified in the Guidance Principles, level of prostatic tenderness, prostate texture, and expressed prostatic secretions tests.
(3) Leukocyte count in prostatic fluid, which is positively correlated with the inflammatory response.^[22]^
(4) Phosphatidylcholine corpuscle count in prostatic fluid, which reflects the treatment effects on sperm liquefaction.^[23]^
(5) Incidence of any adverse event (AE).
(6) Incidence of moxibustion-related AEs.

### 2.5 Search methods for identification of studies

#### 2.5.1 Electronics searches

Nine medical electronic databases will be searched, including PubMed, EMBASE, Cochrane Central Register of Controlled Trials, VIP, Chinese Biomedical Database, China National Knowledge Infrastructure Database, Wanfang Data, Chinese Medicine Database System, and Google Scholar, from the dates of their establishment to March 9, 2019. There will be no restrictions on publication language and publishing status.

#### 2.5.2 Search for other resources

We will search 2 clinical trial registration platforms, Clinicaltrials.gov, and China Clinical Trial Registry, to identify unpublished trials. We will also trace the references of related reviews and check major TCM journals manually.

### 2.6 Search strategy

The search terms will include “prostatitis,” “chronic pelvic pain syndrome,” “prostatodynia,” “moxibustion,” “moxa,” and so on. Text words and medical subject headings will be used as the search fields to devise search strategy. The search strategy to be used in PubMed is as follows: (prostatitis[mh] OR prostatitis[tw] OR prostatodynia[tw] OR prostatalgia[tw] OR chronic pelvic pain syndrome[tw] OR CP[tw] OR CPPS[tw] OR CP/CPPS[tw]) AND (moxibustion[mh] OR moxibustion[tw] OR moxabustion[tw] OR mugwort[tw] OR moxa[tw]) NOT (animals[mh] NOT humans[mh])

### 2.7 Data collection and analysis

#### 2.7.1 Selection of studies

**Figure 1:**
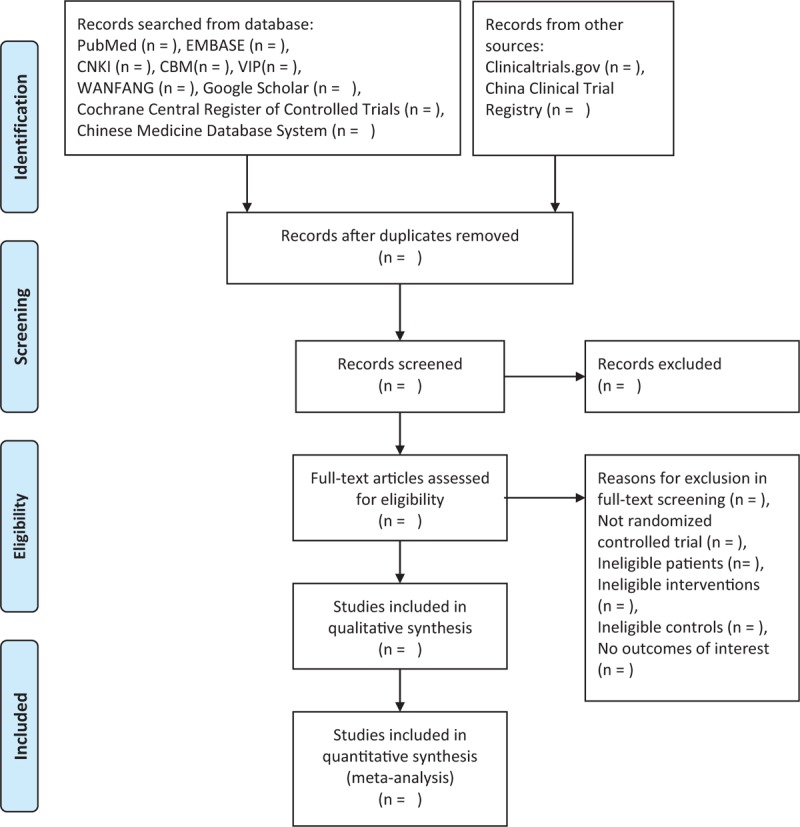
Flow diagram of study selection.

The bibliographies yielded by the literature search will be imported into EndNote X9 (Clarivate Analytics US LLC, Philadelphia) for management. Two reviewers will independently read the literature titles, abstracts, and full texts, in sequence, to identify eligible RCTs. Inter-reviewer inconsistency will be resolved by discussion or by a third party's arbitration. The planned selection process is shown in a PRISMA-P flow chart (Fig. 1).

#### 2.7.2 Data extraction and management

Two reviewers will independently extract the following data according to a pre-designed data extraction form.

(1) Study characteristics: authors’ name, publication year, country, language, sample size, inclusion criteria, type and details of moxibustion, type of control, number of patients, follow-up period, and attrition rate.
(2) Patient characteristics: sex, age, comorbidities, and baseline NIH-CPSI score.
(3) Results data: mean and standard deviation of continuous outcomes and number of events and total population of binary outcomes.

#### 2.7.3 Assessment of risk of bias

The risk of bias of individual RCTs will be assessed using the risk of bias tool in the Cochrane Handbook 5.1.0. The indicators of risk of bias include random sequence generation, allocation concealment, blinding of patients and caregivers, blinding of outcome assessment, data completeness, selective outcome reporting, and other biases. Each indicator will be judged as “yes” (low risk of bias), “no” (high risk of bias), and “unclear” (lack of relevant information or uncertain bias). Additionally, we will provide a further judgment of “probably yes” or “probably no” for the results with unclear risk of bias. Two reviewers will independently complete the risk of bias assessment. Cases of disagreement will be resolved through discussion or will be judged by a third reviewer.

#### 2.7.4 Measures of treatment effect

Mean difference (MD) and 95% confidence intervals (CIs) will be used as the effect measures of continuous outcomes. For binary outcomes, we will use risk ratio (RR) and 95% CIs as the effect measures.

#### 2.7.5 Unit of analysis issues

If there are 2 or more moxibustion groups or control groups, the congeneric results will be merged. If a study provides data of multiple follow-up nodes, we will only use the final data for data analysis. In crossover trials, only the first phase data will be used.

#### 2.7.6 Dealing with missing data

We will estimate missing means and standard deviations of change from baseline in continuous outcomes, using the baseline and last follow-up data according to the Cochrane Handbook's methods.^[24]^ We will contact the corresponding authors via email to request any missing or incomplete data if needed.

#### 2.7.7 Assessment of heterogeneity

Heterogeneity will be estimated by the Cochran's Q test and the I^2^ statistics. When the *P* value in the Q test is >0.10 and an I^2^ is <50%, heterogeneity will be considered as low. Conversely, heterogeneity will be considered as high. A random-effects model will be used to synthesize data in all meta-analyses, regardless of the magnitude of the statistical heterogeneity.

#### 2.7.8 Assessment of reporting biases

In order to detect publication bias, funnel plots will be drawn for the outcomes in which more than 10 studies are included.^[25]^ Harbord test and Egger test will be used for quantitative assessments of publication bias for binary and continuous outcomes, respectively. A *P* value <.05 will indicate significant publication bias. Stata v14.0 (Stata Corp Lp, TX) will be used to perform tests for publication bias.

#### 2.7.9 Data synthesis

We will perform meta-analysis for 2 independent scenarios:

1) add-on comparison (i.e., moxibustion + drugs versus drugs) and
2) active comparison (i.e., moxibustion vs drugs).

RR and 95% CIs will be pooled for the binary outcomes using the Mantel–Haenszel method, and weighted mean difference (WMD) and 95% CIs will be pooled for continuous data using the inverse variance method.^[26]^ If units of continuous data cannot be converted to be unified, standardized mean difference (SMD) will be calculated and pooled in the meta-analysis. If there is such substantial heterogeneity among RCTs that it would be inappropriate to perform meta-analysis, we will provide a narrative summary. We will use forest plots to present the results of meta-analysis. RevMan v5.3 (The Cochrane Collaboration, The Nordic Cochrane Centre, Copenhagen, Denmark) will be used for meta-analysis.

##### 2.7.9.1 Subgroup analysis and investigation of heterogeneity

We will investigate whether heterogeneity is caused by the following confounders through subgroup analysis:

(1) Type of moxibustion: direct moxibustion versus indirect moxibustion.
(2) Course of disease: <1 year versus ≥1 year.
(3) Length of follow up: <3 months versus ≥3 months.

##### 2.7.9.2 Sensitivity analysis

To evaluate the robustness of the results, a second meta-analysis will be conducted by excluding articles with high risk of bias.^[27]^ We will also use a substitutive effect model (random-effects model vs fixed-effects model) for sensitivity analysis.

### 2.8 Ethics and dissemination

This study does not require ethical approval because no personal data will be involved. We will disseminate the results by publishing this systematic review in a peer-reviewed journal.

## 3 Discussion

To our knowledge, no previous systematic review, designed to assess the efficacy and safety of moxibustion treatment for CP/CPPS, has been reported. A systematic review of acupuncture treatment for CP/CPPS was published in 2016.^[28]^ This review assessed electroacupuncture, warming acupuncture, abdominal acupuncture, and auricular acupuncture. However, these acupuncture treatments differ greatly from moxibustion in CP/CPPS treatment. The treatment mechanism of moxibustion for CP/CPPS mainly depends on heat stimulation of vessels and tissues, while acupuncture achieves analgesia and detumescence in CP/CPPS through needling stimulation of postganglionic sympathetic fibers, improving the release of catecholamines and endogenous opioid peptides.^[29]^ Moreover, because of its complex manipulation and requirement for highly sanitary conditions, acupuncture can only be implemented by certified clinicians. Conversely, moxibustion is generally non-invasive and patients can perform moxibustion by themselves or with the help of family members, which substantially enhances the accessibility of moxibustion and patient compliance. Therefore, the findings of the systematic review on acupuncture treatments cannot be applied mechanistically to answer specific questions related to moxibustion treatment for CP/CPPS.

There are some potential limitations in this systematic review. First, we will not set restrictions on publication language in the literature searches. Databases in languages other than English and Chinese, such as Japanese and Korean databases, cannot be involved because of language gaps, which may cause language bias. Second, it is expected that there will be not so many published studies on moxibustion treatment; thus, there may be insufficient samples to perform some additional analyses, such as subgroup analysis and tests for publication bias. Nevertheless, based on the appropriate study design and rigorous assessment, we anticipate that this review will provide critical evidence of moxibustion treatment for CP/CPPS for practitioners and healthcare policy-makers.

## Author contributions

**Conceptualization:** Xu Zhou, Yong Fu.

**Funding acquisition:** Xu Zhou, Yong Fu.

**Investigation:** Jianrong Chen, Weifeng Zhu, Heyun Nie, Meilu Liu, Haifeng Zhang, Yuting Zhong.

**Methodology:** Qianan Cao, Qi Ao, Weifeng Zhu.

**Writing – original draft:** Qianan Cao, Xu Zhou.

**Writing – review & editing:** Yong Fu, Xu Zhou.

## References

[1] Rees J Abrahams M Doble A Diagnosis and treatment of chronic bacterial prostatitis and chronic prostatitis/chronic pelvic pain syndrome: a consensus guideline. BJU Int 2015;116:509–25. 25711488 10.1111/bju.13101 PMC5008168

[2] Suskind AM Berry SH Ewing BA The prevalence and overlap of interstitial cystitis/bladder pain syndrome and chronic prostatitis/chronic pelvic pain syndrome in men: results of the RAND Interstitial Cystitis Epidemiology male study. J Urol 2013;189:141–5. 23164386 10.1016/j.juro.2012.08.088 PMC3894747

[3] Liang CZ Li HJ Wang ZP The prevalence of prostatitis-like symptoms in China. J Urol 2009;182:558–63. 19524948 10.1016/j.juro.2009.04.011

[4] Giannantoni A Porena M Gubbiotti M The efficacy and safety of duloxetine in a multidrug regimen for chronic prostatitis/chronic pelvic pain syndrome. Urology 2014;83:400–5. 24231216 10.1016/j.urology.2013.09.024

[5] Lee CB Ha US Lee SJ Preliminary experience with a terpene mixture versus ibuprofen for treatment of category III chronic prostatitis/chronic pelvic pain syndrome. World J Urol 2006;24:55–60. 16418872 10.1007/s00345-005-0039-x

[6] Nickel JC Narayan P McKay J Treatment of chronic prostatitis/chronic pelvic pain syndrome with tamsulos: a randomized double blind F trial. J Urol 2004;171:1594–7. 15017228 10.1097/01.ju.0000117811.40279.19

[7] Holt JD Garrett WA McCurry TK Common questions about chronic prostatitis. Am Fam Physician 2016;93:290–6. 26926816

[8] Huang C Liang J Han L Moxibustion in early Chinese medicine and its relation to the origin of meridians: a study on the unearthed literatures. Evid Based Complement Alternat Med 2017;2017:8242136. 28298936 10.1155/2017/8242136 PMC5337347

[9] Qi Q Liu YN Jin XM Moxibustion treatment modulates the gut microbiota and immune function in a dextran sulphate sodium-induced colitis rat model. World J Gastroenterol 2018;24:3130–44. 30065559 10.3748/wjg.v24.i28.3130 PMC6064969

[10] Liangyue D Yijun G Shuhui H Chinese acupuncture and moxibustion. Traditional Medicine in Asia 2001 75.

[11] Lee MS Choi TY Kang JW Moxibustion for treating pain: a systematic review. Am J Chin Med 2010;38:829–38. 20821815 10.1142/S0192415X10008275

[12] Liu Y Chen W Tan Y Analysis of the registration information on interventions of acupuncture and moxibustion trials in the international clinical trials registry platform. Evid Based Complement Alternat Med 2018;2018:1054629. 29552076 10.1155/2018/1054629 PMC5820559

[13] Khan FU Ihsan AU Khan HU Comprehensive overview of prostatitis. Biomed Pharmacother 2017;94:1064–76. 28813783 10.1016/j.biopha.2017.08.016

[14] Yu Y Kang J Clinical studies on treatment of chronic prostatitis with acupuncture and mild moxibustion. J Tradit Chin Med 2005;25:177–81. 16334718

[15] Xiao A Chen R Kang M Heat-sensitive moxibustion attenuates the inflammation after focal cerebral ischemia/reperfusion injury. Neural Regen Res 2012;7:2600–6. 25368636 10.3969/j.issn.1673-5374.2012.33.005 PMC4200727

[16] Huang TR Li W Peng B Correlation of inflammatory mediators in prostatic secretion with chronic prostatitis and chronic pelvic pain syndrome. Andrologia 2018;50:e12860. 10.1111/and.12860 28762547

[17] Li ZY Yang YT Hong J Extracellular signal-regulated kinase, substance P and neurokinin-1 are involved in the analgesic mechanism of herb-partitioned moxibustion. Neural Regen Res 2017;12:1472–8. 29089993 10.4103/1673-5374.215259 PMC5649468

[18] Shamseer L Moher D Clarke M Preferred reporting items for systematic review and meta-analysis protocols (PRISMA-P) 2015: elaboration and explanation. Bmj 2015;350:g7647. 25555855 10.1136/bmj.g7647

[19] Krieger JN Nyberg L Jr Nickel JC NIH consensus definition and classification of prostatitis. Jama 1999;282:236–7. 10422990 10.1001/jama.282.3.236

[20] Propert KJ Litwin MS Wang Y Responsiveness of the National Institutes of Health Chronic Prostatitis Symptom Index (NIH-CPSI). Qual Life Res 2006;15:299–305. 16468084 10.1007/s11136-005-1317-1

[21] Zheng X Guiding principle of clinical research on new drugs of traditional Chinese medicine. 2002;Beijing, China: China Medic-Pharmaceutical Sciences and Technology Publishing House, 143.

[22] Zdrodowska-Stefanow B Ostaszewska-Puchalska I Badyda J The evaluation of markers of prostatic inflammation and function of the prostate gland in patients with chronic prostatitis. Arch Immunol Ther Exp (Warsz) 2008;56:277–82. 18661262 10.1007/s00005-008-0031-4 PMC2778713

[23] Guo J Li C Wu B Tetracycline treatment of type III prostatitis nanobacteria infection of 100 cases report. Paper Present AIP Conference Proc 2018;1956:020030.

[24] Lefebvre C Manheimer E Glanville J Higgins JP Green S Searching for studies. Cochrane Handbook for Systematic Reviews of Interventions. Chichester, UK: John Wiley & Sons, Ltd; 2008 188–235.

[25] Song F Gilbody S Bias in meta-analysis detected by a simple, graphical test. Increase in studies of publication bias coincided with increasing use of meta-analysis. BMJ 1998;316:471. PMC2665616 9492690

[26] Faraone SV Interpreting estimates of treatment effects: implications for managed care. P t 2008;33:700–11. 19750051 PMC2730804

[27] Higgins JP Altman DG Gotzsche PC The Cochrane Collaboration's tool for assessing risk of bias in randomised trials. Bmj 2011;343:d5928. 22008217 10.1136/bmj.d5928 PMC3196245

[28] Qin Z Wu J Zhou J Systematic review of acupuncture for chronic prostatitis/chronic pelvic pain syndrome. Medicine (Baltimore) 2016;95:e3095. 26986148 10.1097/MD.0000000000003095 PMC4839929

[29] Lee SH Lee BC Use of acupuncture as a treatment method for chronic prostatitis/chronic pelvic pain syndromes. Curr Urol Rep 2011;12:288–96. 21472420 10.1007/s11934-011-0186-0

